# Impact of an oral health education intervention among a group of patients with eating disorders (anorexia nervosa and bulimia nervosa)

**DOI:** 10.1186/s40337-019-0259-x

**Published:** 2019-09-05

**Authors:** Laura S. Silverstein, Carol Haggerty, Lattice Sams, Ceib Phillips, Michael W. Roberts

**Affiliations:** 1 Department of Pediatric Dentistry, Children’s Hospital Colorado, 1575 N Wheeling Street, Aurora, CO 80045 USA; 2 Division of Comprehensive Oral Health, University of North Carolina Adams School of Dentistry, Chapel Hill, NC 27599-7450 USA; 3 Division of Craniofacial and Surgical Sciences, University of North Carolina Adams School of Dentistry, Chapel Hill, NC 27599-7450 USA; 4 Division of Pediatric and Public Health, University of North Carolina Adams School of Dentistry, 228 Brauer Hall CB #7450, Chapel Hill, NC 27599-7450 USA

**Keywords:** Oral health education, Eating disorders, Anorexia nervosa and bulimia nervosa

## Abstract

**Background:**

It is recognized that eating disorders are serious psychosocial illnesses that affect many adolescents and adults. A pre and post survey study was developed to assess demographics, oral health knowledge and self-image of patients with eating disorders participating in a hospital-based eating disorder clinic using an original oral health education program. The program’s aim is to change the self-image and oral health practices of patients with anorexia-binge eating/purging (AN-BP) and bulimia nervosa (BN) disorders.

**Methods:**

A pre-survey was completed by each study participant prior to attending the three educational sessions over a six-week period. A post survey questionnaire was completed after participation in all the educational presentations. Forty-six patients attended all three educational sessions and completed the pre and post-questionnaires.

**Results:**

Most patients knew in advance that AN-BP and BN behavior can cause erosion of the teeth but only 30% knew the most likely location for the erosion to occur. But, following completion of the educational interventions, 73% answered the location correctly. Patients who reported going to the dentist regularly were significantly more likely to respond that their teeth/mouth had a positive effect on how they looked to themselves and to others, their general health, and their general happiness. Positive responses to the effect of the teeth/mouth on kissing and romantic relationships were also significantly higher for those who go to the dentist regularly compared to those who do not.

**Conclusions:**

There is a need to further understand AN-BP and BP patients’ oral health knowledge and self-image perceptions as it relates to their smile (teeth, mouth) to assist in developing a standardized oral health program for eating disorder centers to implement into their daily curricula. A dental team member in an interdisciplinary eating disorder treatment team is important. Including an oral health education program improves patients’ oral hygiene and oral health knowledge, as well as provides a supportive environment to empower the patients to take control of their overall oral health.

**Trial registration:**

This study was retrospectively registered on April 18, 2019 in ClinicalTrials.gov, Identifier: NCT03921632.

## Plain English summary

Using a pre and post survey, this study examined the demographics, oral health knowledge and self-image among a group of patients diagnosed with AN-BP or BN. The study participants completed a survey instrument before attending three educational presentations. A post survey questionnaire was completed after the final educational presentation. After participating in the educational presentations, 95% knew that tooth erosion was the most common oral effect of eating disorders (pre education = 78%) and 73% knew where the erosion most commonly occurred (pre education = 30%). Patients who went to a dentist regularly were significantly more likely to report that their teeth/mouth had a positive effect on how they looked to themselves and others, romantic relationships, general health, and their level of happiness. Providing an oral health education program improves the patients’ oral health knowledge and empowers them to be proactive in caring for their teeth.

## Introduction

Eating disorders are psycho-social illnesses that affect many adolescents and adults [1]. Individuals with eating disorders can also have additional health issues [2]. Some of these include diabetes, loss of menses in females, heart failure, very low self- esteem, metabolic, cardiovascular and endocrine disturbances [2], and distorted perception of body image [3]. In addition to these systemic problems, oral/dental trauma and dental caries, increase in xerostomia and parotid salivary gland swelling, dental erosion, and periodontal disease have been cited in the literature and are most associated with anorexia-binge eating/purging (AN-BP and bulimia nervosa (BN) [4]. Roberts and Li stated that the very negative self-perception and self-esteem that many individuals with anorexia nervosa and bulimia nervosa reported could be, in part, a cause for their lack of oral hygiene and increase in dental disease [3].

Often there are general health education programs in eating disorder clinics. However, we are unaware of any established oral health education programs to change and improve patients’ oral health behaviors and self-perception as it relates to their smile [5, 6]. According to the Academy of Eating Disorders, the American Psychological Association, and the American Dental Association, proper oral health care for patients with eating disorders is fragmented. Johnson et al. reported “In addition to patient’s lack of knowledge, there is very little evidence, if any, documenting the collaborative interaction between oral health and eating disorder professionals. Individuals who receive treatment for an eating disorder are typically not viewed as needing specialized, preventive oral care and commonly fail to receive appropriate oral health care, or recommendations from the eating disorder treatment team” [7]. There is no indication that these patients have access to the proper oral health education and information. Without adequate oral health education for patients with eating disorders, oral disease can become severe and impact the patient’s ability to eat, speak, and socialize [7].

The extent of oral/dental education in curricula within eating disorder treatment centers is unexplored to date [5, 6, 8]. Evidence of patients’ self-image as it relates to their smile is also unexplored. The limited studies evaluating perception of oral health knowledge in eating disorder treatment programs suggest the need to further explore provider and client/patient barriers and perception towards oral care [7]. Integrating an oral health program focusing on improving self-image through dental education is essential in providing patients an interdisciplinary approach to care for the entire person. Understanding eating disorder patients’ oral health knowledge and perceptions of self-image as it relates to their smile will assist in developing a standardized oral health program for eating disorder treatment centers to implement into their daily curricula.

## Methods

**Fig. 1:**
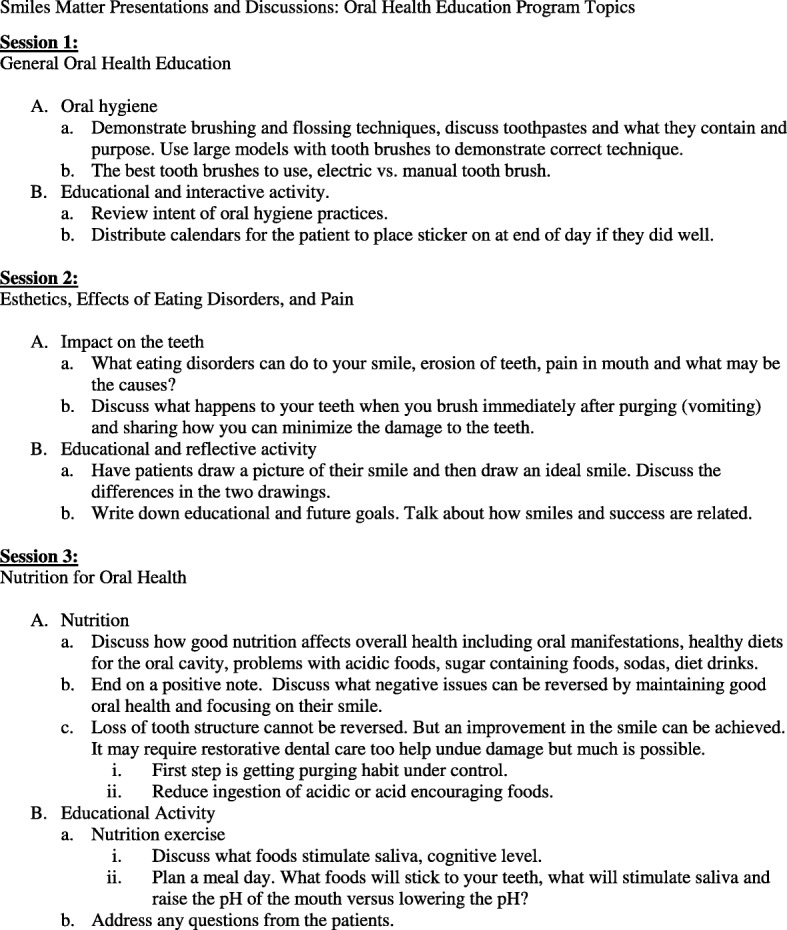
Smiles Matter Presentations and Discussions: Oral Health Education Program Topics

The authors designed a pre and post survey study to assess demographics, oral health knowledge, and self-image of patients diagnosed with AN-BP and BN participating in a hospital-based eating disorder clinic. The authors consulted and collaborated with eating disorder specialists, dentists, dental hygienists, and psychiatrists in designing the surveys and the education program. An original oral health education program, called Smiles Matter, was created consisting of three different presentations/discussions. Each Smiles Matter session consisted of 15–20 min of didactic learning, 10 min of a group/personal exercise, and 10 min of questions that the patients had about their oral health or the topic of the day (Fig. 1).

Patients were consented prior to participating in the study and educational program. A pre-survey was completed by each participant prior to attending the three educational sessions. The presentations were given weekly and addressed general oral health education, esthetics, effects of eating disorders and oral pain, and nutrition for oral health. A post survey questionnaire was completed after the patient had participated in all three educational presentations. A respondent could choose not to answer all questions and some did so. Therefore, the denominator for some questions is different depending upon the number of responses.

The pre-survey consisted of 37 questions and the post survey consisted of 17 questions. Copies of the survey instruments are available from the author. The pre-survey asked questions on the following topics: demographics, oral health knowledge, oral habits and oral health behaviors prior to diagnosis, oral health and habits since diagnosis with AN-BP or BN and current self-perceptions. The post-survey addressed similar issues after the patients participated in the education program. Chi-square analyses were performed to compare those who completed only the pre-survey questionnaire and those who completed both surveys and to assess the effect of age (< 23 vs > =23) and dental visit frequency (regularly vs occasionally/if a problem) on the pre-survey self-perception items. Discordance between pre and post survey responses were analyzed using the McNemar Test or its extension, the Bowken’s Test of Symmetry. All analyses were conducted using SAS®v9.4software. Level of significance was set at 0.05 for all analyses. Statistical results were not corrected for multiplicity of comparisons because of the nature of the study.

The study design and all questionnaires, presentations, consents to participate and hand-outs were reviewed and approved by the Institutional Review Boards (IRB) at the University of North Carolina at Chapel Hill (IRB #15–3295). All study sites ceded review responsibility to the UNC-Chapel Hill IRB for management. The study is registered with ClinicalTrials.gov (Identifier: NCT03921632). Funding for this study was provided by the Department of Pediatric Dentistry.

### Study population

Patients enrolled in three in-patient treatment programs participated in the study; Center of Excellence for Eating Disorders at the University of North Carolina School of Medicine, Carolina House Eating Disorder Treatment Center, and Veritas Collaborative. Patients between the ages of 13 years old and 50 years old were eligible to participate in the study. While there are now eight recognized classifications of eating disorders in the DSM V [9], this study was limited to patients with anorexia nervosa and bulimia nervosa. Most of the patients who participated in the study did classify themselves as having anorexia nervosa.

## Results

Sixty-seven patients were initially screened (92% Caucasian/100% females) and completed the pre-survey questionnaire but only 46 completed both pre- and post- questionnaires and attended all three of the educational module presentations. This was due to the patient being released from the clinic or problems with scheduling return visits. There were no statistically significant differences between those who completed the protocol and those who did not (Table 1).

**Table 1:** Comparison of those who participated in both the pre and the post survey (*N* = 46) and those who only participated in the pre-survey (*N* = 21)

Variable	Participated in Both Surveys			Completed Only Pre-Survey			*P* Value
	Mean		SD	Mean		SD	
Age at Entry	25.2		10.8	26.9		10.7	0.55
Age at Diagnosis	21.4		10.3	20.6		8.2	0.77
	N	%		N	%		
Eating Disorder							0.77
Anorexia nervosa	31	69		13	62		
Bulimia Nervosa	10	22		5	24		
Other	4	9		3	14		
Ever Seen a Dentist							0.79
No	1	2		0	0		
Within last year	27	59		15	71		
Last 2 years	10	22		3	14		
More than 2 years	8	17		3	14		
Frequency of Dental Visit							0.93
Regularly	27	59		13	65		
Occasionally	10	22		4	20		
Only if problem	9	20		3	15		

Of those who completed the protocol, the mean age at the time of diagnosis was 21.4 years (SD = 10.3) and at the beginning of the study 25.2 years old (SD = 10.8). The majority (69%) of the patients had AN-BP. Fifty-nine percent reported seeing a dentist regularly but 20% reported seeing a dentist only when they had a dental problem. Only 15% of the patients reported being referred to a dentist since their eating disorder was diagnosed. While most patients knew in advance eating disorders behavior can cause erosion of the teeth (*N* = 35; 76%) only 30% (*N* = 14) knew the most likely location in the mouth for erosion to occur. Eight-8 % also reported tooth sensitivity as an oral effect of an eating disorder; 78% reported dry mouth as an effect while 57% thought salivary gland enlargement and 38% oral cancer were possible oral effects (Table 2).

**Table 2:** Effect of dental visit frequency on correct identification of possible oral effects of eating disorders and positive self-perception before the intervention

Possible effects of eating disorders	Regularly		Occasionally/only if problem		*P* value
	N	%	N	%	
Tooth erosion	22 of 26	84	13 of 20	65	0.45
Probable erosion sites	10 of 26	38	4 of 20	20	0.33
Tooth sensitivity	24 of 26	92	14 of 20	70	0.37
Dry mouth	20 of 26	81	13 of 20	66	0.99
Enlarged saliva glands	16 of 26	62	8 of 20	40	0.53
Oral cancer	12 of 26	46	4 of 20	20	0.21
No pain	14 of 26	54	7 of 20	35	0.02
Positive self-perception responses					
Confidence	15 of 26	58	5 of 20	25	0.15
Look to others	15of 26	58	3 of 20	15	0.03
Kissing	12 of 26	46	2 of 20	10	0.04
General health	17 of 26	65	5 of 20	25	0.01
Attendance	7 of 26	27	2 of 20	10	0.54
Success	7 of 26	27	4 of 20	20	0.60
Smiling/laughing	19 of 26	73	7 of 20	35	0.12
Looks to themselves	15 of 26	58	4 of 20	20	0.03
Social life	10 of 26	37	3 of 20	15	0.18
Enjoy eating	14 of 26	54	6 of 20	30	0.27
Speech	14 of 26	54	5 of 20	25	0.10
Choice of foods	8 of 26	30	4 of 20	20	0.17
Enjoy Life	14 of 26	54	3 of 20	15	0.08
Romantic relationship	10 of 26	37	1 of 20	5	0.002
General happiness	11 of 26	42	0 of 20	0	< 0.001
Weight	4 of 26	15	2 of 20	10	0.37

Before the intervention, there were no statistically significant differences (*p* > 0.06) in the proportion of positive responses for those less than 23 years of age (54%) compared to those 23 or older (46%) with respect to the effect that the teeth / mouth have on self-perception. Patients who reported going to the dentist regularly were significantly more likely to respond that their teeth/mouth had a positive effect on how they looked to themselves (*p* = 0.03), how they looked to others (*p* = 0.03), their general health (*p* = 0.01), and their general happiness (*p* < 0.001) than those who only reported going occasionally, or if they had a problem. Positive responses to the effect of the teeth/mouth on kissing and romantic relationships were also significantly higher for those who go to the dentist regularly compared to those who don’t (*p* = 0.04 and 0.002 respectively). Table 2. Sixty-three percent (*N* = 27) of the patients said they had a plan to use the Smiles Matter material. Only 11% (*N* = 5) did not plan to see a dentist or would go only if they had a problem while 67% (*N* = 30) planned to see a dentist within 6 months. After participating in the program, 95 % of the patients (*N* = 41) correctly identified dental erosion as the most common dental finding of eating disorders. Similarly, 73% (*N* = 32) after the program answered correctly where erosion is most likely to occur in the mouth (Table 3).

**Table 3:** Impact of “Smiles Matter” program

Plan to See a Dentist	N		%			
No	2		4			
Within 6 months	30		67			
Within 1 year	10		22			
Only if a problem	3		7			
Have a Plan to Use Smiles Matter						
No	16		37			
Yes	27		63			
Change in Knowledge						
Dental Erosion						
	Post “Smiles Matter”					
	Correct		Incorrect			
Pre “Smiles Matter”	N	%	N	%		*P* value
Correct	34	79	1	2.3	(*N* = 35)	0.03
Incorrect	7	16	1	2.3		
	(*N* = 41)					
Erosion Location						
Correct	12	27	2	4.6	(*N* = 14)	0.001
Incorrect	20	45	10	22.7		
	(*N* = 32					

## Discussion

Evidence-based communication programs are critical for health professionals managing the prevention, treatment, and post treatment of patients of patients with AN-BP and BN eating disorders. It is important that oral health education programs be included to assist eating disorder patients improve their self-image and shift their focus to the importance of their smile and oral health. The results of this study suggests improved communication and providing appropriate information to AN-BP and BN eating disorder patients will help change behaviors and improve their oral health. The intent of this study was to survey the effectiveness of an original oral health education program, “Smiles Matter”, which aimed to improve patient’s oral hygiene practices and oral health knowledge, and provide a supportive environment to empower them to take control of their oral health as well as their general health. Throughout the study, participating patients verbally shared that they had not previously had specialized, preventive oral care or recommendations from their eating disorder treatment team targeted to oral health.

It is important that eating disorder patients have access to a dental home to help provide a supportive environment for their oral health, their self-image as it relates to their smile, and their general health. The study found that patients who went to the dentist more frequently had a more positive response to how their teeth affected their lives. It is important for eating disorder treatment centers to provide an oral health educational program and to include an oral health educational program in their treatment protocol.

The primary limitation of this study was the small sample size. It was a challenge to identify patients who were available to complete all components of the study due to the intensity, length, and location of the eating disorder treatment programs. The dropout rate (patients not completing the entire program) was another limiting factor. While additional history was probably recorded in the patient’s clinical record (e.g. duration of illness, psychiatric comorbidity) this information was not recorded/analyzed in the present study.

## Conclusions

While statistical significance were shown in this study, the need to further understand eating disorder patients’ oral health knowledge and perceptions of self-image as it relates to their smile (teeth, mouth) is important to assist in developing a standardized oral health program for eating disorder treatment centers to implement into their daily curricula/protocol. Including an oral health education program in eating disorder treatment centers appears to improve patients’ oral hygiene and oral health knowledge, as well as provides a supportive environment to empower the patients to take control of their overall oral health. Whether the initial improvement in oral habits and awareness will be maintained over time could not be determined by this study.

## Data Availability

The data supporting the results reported in this article is maintained in the UNC Adams School of Dentistry (UNC-ASOD), Department of Pediatric Dentistry, Chapel Hill, North Carolina USA. Please contact author for data requests.

## Abbreviations

AN_BP: Anorexia-binge eating/purging
BN: Bulimia nervosa
IRB: Institutional Review Board
UNC: University of North Carolina-Chapel Hill
